# Mobile Phone Apps for Behavioral Interventions for At-Risk Drinkers in Australia: Literature Review

**DOI:** 10.2196/mhealth.6832

**Published:** 2018-02-13

**Authors:** Carol C Choo, André A D Burton

**Affiliations:** ^1^ College of Healthcare Sciences James Cook University Singapore Singapore; ^2^ School of Psychology Murdoch University Perth Australia

**Keywords:** problem drinking, alcohol drinking, eHealth, telemedicine, smartphone, mobile applications, behavioral intervention, risk reduction behavior, review

## Abstract

**Background:**

The mobile technology era has ushered in the use of mobile phone apps for behavioral intervention for at-risk drinkers.

**Objective:**

Our objective was to review recent research relevant to mobile phone apps that can be used for behavioral intervention for at-risk drinkers in Australia.

**Methods:**

The inclusion criteria for this review were articles published in peer-reviewed journals from 2001 to 2017 with use of the search terms “smartphone application,” “alcohol,” “substance,” “behavioural intervention,” “electronic health,” and “mobile health.”

**Results:**

In total, we identified 103 abstracts, screened 90 articles, and assessed 50 full-text articles that fit the inclusion criteria for eligibility. We included 19 articles in this review.

**Conclusions:**

This review highlighted the paucity of evidence-based and empirically validated research into effective mobile phone apps that can be used for behavioral interventions with at-risk drinkers in Australia.

## Introduction

In Australia, alcohol is a common substance of dependence for which individuals seek treatment [1]. Reducing the risk of alcohol-related harm is important in Australia, with a government that takes a harm minimization approach [2-4]. The preferred approach for alcohol interventions in Australia has been to prevent the adverse consequences associated with alcohol consumption rather than banning drinking altogether [5]. Psychological interventions informed by the stages of change model [6,7], as well as therapeutic techniques from motivational interviewing [8], cognitive behavioral approaches [9], and self-management strategies [8,10], hold promise to change problematic behaviors [7,11] and address adverse consequences related to drinking [12].

In recent decades, the advent of mobile phone technology has transformed the mode of delivery of psychological treatment [13]. Through promotion of the accessibility of interventions via mobile phone apps, alcohol dependence interventions may be enhanced and the adverse consequences of risky drinking may be reduced [6]. The demand for electronic health apps across Australia and the world is mirroring larger societal trends wherein consumer acceptance of technology has grown [14,15]. Community interest has increased in Australia regarding the use of mobile phone apps to address substance abuse [16], health monitoring, and self-management [17]. Some clinics in Australia have implemented conjunctive treatment modalities in guided programs such as cognitive behavioral therapy and psychoeducation apps alongside face-to-face therapy sessions [17]; for example, the DBT Diary Card & Skills Coach [18] was designed as an adjunctive tool to therapy for individuals recovering from substance abuse. However, research examining its effectiveness lacked conclusive evidence due to the lack of distinction made between the different types of substance use [19,20].

Our aim was to review research relating to the evidence for mobile phone apps that can be used for behavioral intervention for at-risk drinkers in Australia.

The literature positions mobile phone apps under the umbrella of mobile health and its subcategory electronic health, which is defined as health care practice supported by electronic processes and communication [21]. For this review, smartphone refers to a mobile phone that performs many of the functions of a computer. This typically includes having a touchscreen interface, Internet access, and an operating system capable of running downloaded apps. A mobile app is a computer program designed to run on mobile devices such as smartphones and tablet computers. It allows for third parties to design software and apps that can then be downloaded by the user at their discretion.

At-risk drinker is defined as a heavy drinker who consumes 5 or more drinks on the same occasion on each of 5 days or more in the past 30 days [22]. In contrast to a binge drinker, who has a pattern of drinking that brings blood alcohol concentrations up rapidly after consuming alcohol in one go, an at-risk drinker displays consistency in their heavy drinking levels.

## Methods

The inclusion criteria for this review were publication in peer-reviewed journals from 2001 to 2017 with use of the search terms “smartphone application,” “alcohol,” “substance,” “behavioural intervention,” “electronic health,” and “mobile health.” The databases we searched were PsycINFO, Scopus, Google Scholar, and PubMed.

We initially used the PsycINFO database to identify peer-reviewed articles with the inclusion criteria named above; this yielded 11 results. The Scopus database search yielded 19 articles. We then conducted hand searches: a backward search using the reference lists of relevant articles and a forward search that checked publications from authors who had cited these relevant articles. The backward and forward searches generated 11 more articles. The focus was on recently published articles in peer-reviewed journals that fit the inclusion criteria and were relevant to a mobile phone app that could be used for behavioral intervention for at-risk drinkers in Australia.

We retrieved articles if they related to interventions provided via a mobile phone app for at-risk drinkers. The strategy for evaluating eligibility for inclusion involved the following: recent articles that contained original work published in peer-reviewed journals after the year 2001; and articles related to use of a mobile phone app by clinicians for therapeutic purposes. We excluded articles that did not refer to the use of mobile phone apps by clinicians for therapeutic purposes.

## Results

A total of 103 articles satisfied all inclusion criteria in the original search across all the databases. Of the original 103 search results, we screened 90 articles, after which we assessed 50 full-text articles against the inclusion and exclusion criteria, and then deemed 19 of these to be suitable for inclusion in this review [17,23-40]. Multimedia Appendix 1 presents the results of the review.

Overall, the articles show a lack of convincing evidence of effective mobile phone apps that can be used for behavioral intervention for at-risk drinkers in Australia. Randomized controlled trials did not yield significant results on the primary outcome [23,24]. Other studies were limited by small sample sizes [25,26] or only reviewed mobile phone apps [27] and did not specifically address our research question [17] of whether the mobile phone app was effective for behavioral intervention for at-risk drinkers in Australia. Although qualitative studies are not typically included in a systematic review, we decided to include these in our table (Multimedia Appendix 1) to illustrate the state of research in Australia, that convincing evidence is still lacking. A study in Australia conducted by Weaver and colleagues [26] reviewed available mobile phone apps and then used a qualitative methodology of focus groups, which offers preliminary exploration. However, it does not offer evidence for their use within the demographic group most at risk for developing alcohol problems in Australia, namely men aged 20 to 29 years and indigenous youths [22,41], who often develop dysfunctional drinking habits that maintain their dependence [42]. Risky drinking in younger demographics is known to be a risk factor for suicidality [43] and other adverse mental health outcomes.

## Discussion

As younger demographics are more likely to access online information relating to mental health problems [44-46], mobile technologies can enhance patient-centered care for youths and young adults in an increasingly technology-savvy society [28], highlighting a growing need to offer electronic interventions as an adjunctive tool to face-to-face therapy [47,48]. Evidence for the use of mobile phone apps has been demonstrated in many other areas [49-54] but not for at-risk drinking in Australia. Internet-based interventions have been found to be efficacious for mental health issues [3] in young adults [45,47,55]. Behavioral monitoring apps have been used for mental health interventions [29,56] in addition to face-to-face therapy. Positive outcomes were shown in overall motivation [57], and in maintaining and reinforcing behavioral changes [16,57,58]. These apps show promise for use with ethnically diverse and low-income populations [59] to enhance support [17], help them to cope, and aid in recovery [60,61]. Behavioral data can be quantified into graphs [56] and used by clinicians [29,62]. However, youths view apps as a form of entertainment rather than therapeutic tools [26]. The focus could be shifted with an emphasis on behavioral modification instead [63] and apps could be used as an adjunctive tool to complement face-to-face therapy delivered by qualified health professionals [2,15,64]. More research is needed to support the effectiveness of such apps for use with indigenous youths and young adults in Australia.

Mobile phone interventions have been used for drinking problems in a few clinics in the United States [29] but with less compelling evidence for clinics in Australia. Behavioral monitoring apps are being used for digital behavior change interventions that provide goal setting and behavior monitoring [30], which also allow for triggers to be detected. The AlcoDroid Alcohol Tracker [65] allows for tracking alcohol consumption, as does the Alcohol Tracker [66]. Most of these apps are based on simple features that estimate the amount of alcohol in the blood [67-70], which could be used to set specific drinking targets but do not constitute the most important element for the monitoring of risky drinking [26].

Despite a large increase in research on electronic interventions in recent years (refer to Multimedia Appendix 1), gaps in knowledge remain. Specifically, there is a lack of strong evidence examining the efficacy of mobile phone apps that have been empirically validated with rigorous scientific methods for at-risk drinkers in Australia, especially young males [4,29] and indigenous youths. Youths can be impressionable consumers, and principles of rigorous scientific inquiry should be applied to explore the benefits of the use of health-related apps in this population [71]. Research aimed at examining low-cost mobile phone apps that are efficacious as an adjunctive tool to therapy would add significantly to the literature [29]. Considering the prevalence of alcohol problems [22], especially in young males and indigenous youths in Australia, research is much needed to explore alternative ways to deliver effective interventions [72].

It is important to understand that any therapy or medical treatment has the potential to cause harm, and that any device can cause adverse effects if used incorrectly. Some critiques of the mobile phone app movement have focused on the ethical importance of protecting consumers from potential harm. There should be laws and regulations [73] governing the operation of mobile phone app stores, and steps should be made available to legislators to protect consumers. This argument follows that if apps were to be used in health care settings for therapy, it is important that the stores be reputable and that the apps be created by legitimate third-party software developers [73]; for example, iTunes App Store currently contains 20,000 apps in the Medical category, yet it is not clear what is precisely relevant for clinical decision making with specific at-risk groups [31].

The critical issue for clinicians using mobile phone apps with their patients is the risk to benefit ratio with such a large selection of apps [73]. During this fledgling stage of exploration when apps are yet to be rigorously assessed and curated formally based on their content, clinicians should carefully consider safety issues. In Australia, it is a prerequisite in the Therapeutic Goods Act [74] for health apps to ensure data security and that all claims made regarding the app comply with the Australian consumer law that they are not misleading the consumer [74]. No apps in Australia fall under the label of medical device, which requires registration under the Therapeutic Goods Act [74]. If apps could be registered as medical devices, perspectives toward privacy may change, since data security would be mandated as a part of the registration [75]. This would also allow regulatory action to be followed through if there were legal issues that needed attention.

There is concern over accessibility in terms of limitations of digital cover in remote communities [75,76]. A difference in network coverage and affordability of the type of mobile phones that can be used to host the app may disadvantage Australians who already experience significant socioeconomic disadvantage and who are also at risk of higher rates of alcohol use [77]. Additionally, a critique has been made on whether youths could become somewhat dependent on apps [78]. However, problem drinkers or those at risk of alcohol addiction are not a homogeneous group, and this must be considered when clinicians are deciding on app suitability for use with their patients. The needs of the patient need to be carefully considered.

In summary, there is consensus that alcohol misuse is a widespread problem in Australia [79]. The health and social consequences resulting from the misuse of alcohol have been widely reported [32,80]. Reducing the risk of alcohol-related harm is important for affected individuals and society at large [2,3]. Enhancing the delivery of interventions may reduce the adverse consequences of alcohol misuse [6]. The potential use of mobile phone apps in the delivery of behavioral interventions tailored for at-risk drinkers remains promising, but evidence to support their use is lacking in Australia. More research is needed to address the gaps in knowledge and to provide an evidence base for the implementation of mobile phone technologies. Developing mobile tools for young users with substance and alcohol abuse issues requires careful ethical consideration regarding the patient-practitioner relationship, the logic of self-surveillance, and overall best practice.

More rigorous research and evaluations are needed to ascertain the efficacy of and establish evidence for best practice for use of such mobile phone apps [17]. The real-time delivery of interventions aimed at reducing risky drinking holds promise to support people who are seeking to change their behavior [32]. Although drinking apps do exist, there are many inconsistencies in their features [26]. Apps that are designed specifically for behavioral interventions for at-risk drinking have not been empirically studied in Australia. Quality and ethical issues relating to the use of such technology need to be considered on a deeper level.

## Appendix

Multimedia Appendix 1 Summary of evidence.
