# Supplementary material for: Mobile Phone Apps for Behavioral Interventions for At-Risk Drinkers in Australia: Literature Review
Source: JMIR Mhealth Uhealth. 2018 Feb 13;6(2):e18. doi: 10.2196/mhealth.6832 (PMC5829458; doi:10.2196/mhealth.6832)
Supplement: Multimedia Appendix 1 [file mhealth_v6i2e18_app1.pdf]

Summary of evidence

Author/Year/Country	Level of Evidence/Study Design/Participants/ Inclusion Criteria	Outcome Measures/Study objectives	Results
Donker, Petrie, Proudfoot, Clarke, Birch, & Christensen (2013)<br><br>*Australia*	[Level I]<br><br>Systematic Review	Primary outcome measures included anxiety, depression, substance use, sleep disturbance, self-harm, suicide ideation as assessed with validated mental health scales.	5464 abstracts were identified in total. 8 papers met the inclusion criteria that described applications targeting anxiety, substance abuse and depression. Four applications provided support from a mental health professional. Results showed significant reductions in substance use (P=.05), stress (P<.001) and depression (P=.03). Within-group and between-group intention-to-treat effect sizes ranged from 0.29-2.28 and 0.01-0.48 at posttest and follow-up
Weaver, Horyniak, Jenkinson, Dietze, & Lim (2013)<br><br>*Australia*	[Level V]<br><br>Applications reviewed followed by qualitative study<br>*N*= 12 young adults (5 males and 7 females).	Primary outcome included young adults' opinions of alcohol-related applications.	A content analysis of 500 smartphone applications was conducted and 384 applications were included. Smartphone applications were effective in managing long-term illnesses and for people suffering from alcohol dependence by providing resources, support and information.
Lathia, Pejovic, Rachuri, Musolesi, & Rentfrow (2013)<br><br>*United Kingdom*	[Level IV]<br><br>(Applications reviewed only)	Objective included exploring behaviour-change interventions and mobile sensing technology	Smartphones can be used for mobile-based behaviour change applications to support behavioural therapy and deliver feedback to clients and clinicians.
Wiechmann, Kwan, Bokarius & Toohey (2016)<br><br>*United States of America*	[Level IV]<br><br>(Applications reviewed only)	Objective included exploring clinical relevance of smartphone apps. Reviewed apps that provided knowledge for clinical decision-making	Out of 7,699 applications, 4,994 applications (64.9%) were considered not relevant to medical professionals. In total, 1,372 (17.8%) were clinical, 738(9.6%) were based on a book or publication, 126 (1.6%) were non-English, 55 (0.7%) were clinically relevant patient

			education resources, and 372 (4.8%) were study tools.<br><br>Results indicated that at this stage clinically relevant applications represent only a small percentage of the total applications in the store.
Aguilera & Muench (2012)<br><br>*United States of America*	[Level V]<br><br>Literature Review	Reviewed use of smartphone applications as an adjunct to clinical care	The majority of Cognitive Behavioural Therapy practitioners have not been using available web-based and mobile applications as adjunctive to clinical care.
Seko, Kidd, Wiljer, & McKenzie (2014)<br><br>*Canada*	[Level I]<br><br>Systematic Review	Objective included review of knowledge regarding mobile mental health for youths (aged 13-24 years)	57 citations were based on broad relevance to the topic area. 19 articles were selected for full text review. Finally, 17 studies were analysed. Results indicated that clinicians should carefully consider ethical issues and best practice.
Hollis,Falconer, Martin,Whittington, Stockton, Glazebrook, & Davies (2017)<br><br>*United Kingdom*	[Level I]<br><br>Systematic and meta-review	Objective included evaluation of the evidence base for digital health interventions	21 reviews were included in the meta-review. The study found support for computerised cognitive behavioural therapy. Methodological limitations made it difficult to draw conclusions. Study found that there was a poor specification of the level of human support, lack of an agreed typology, as well as small sample sizes.
Mellentin, Stenager, Nielsen, Nielsen, & Yu (2017)<br><br>*Denmark*	[Level V]<br><br><br><br>(Applications reviewed only)	Objective included description of design and development of a smartphone application as an innovative delivery pathway for treating alcohol use disorder	The application is currently being tested at a randomized controlled trial.
Ford, Alagoz, Dinauer, Johnson, Pe-Romashko, & Gustafson. (2015)<br><br>*United States of America*	[Level NA]<br><br>Qualitative study<br>*N*=76	Objective included identification of challenges associated with the application A-CHESS that is specifically designed for addiction recovery	The study suggests that successful strategies used by clinicians to sustain A-CHESS usage by patients included leadership support and use of patient feedback reports to follow up with disinterested users.

			Further strategies for engaging both staff and patients in ongoing use of the applications are needed.
Crane, Garnett, Brown, West, & Michie (2015)<br><br>*United Kingdom*	[Level V]<br><br><br>(Applications reviewed only)	Objective included review of alcohol-related applications available in the United Kingdom	800 applications were identified. Behaviour change theory/technique was not found in any of the applications.
Milward, Khadjesari, Fincham-Campbell, Deluca, Watson, & Drummond (2016)<br><br>*United Kingdom*	[Level V]<br><br><br>Applications reviewed followed by qualitative study	Objective included exploring user perspectives regarding the design of a smartphone application and operational features and content that would help to reduce harmful drinking in young adults	1584 applications were extracted. Of these, 201 were classified as alcohol reduction, 154 as Blood Alcohol Content calculators, 509 as entertainment, and 720 as other. Additionally, 32 applications were identified in the study for electronic screening and brief intervention. Brief intervention applications helped to reduce weekly alcohol consumption when delivered by a computer.
Thomas, Linderoth, Bendtsen, Bendtsen, & Mussener (2016)<br><br>*Sweden*	[Level NA]<br><br>Qualitative study<br>*N*=20 (Students *n*=15, and experts *n*=5)	Aimed to develop text message-based intervention targeting harmful alcohol usage among university students	The study found that students preferred short messages and that a 6-week intervention was an appropriate duration. The findings showed positive attitude toward receiving support through text message which were clear and promoted engagement.
Suffoletto, Kristan, Mecca, Chung, & Clark (2016)<br><br>*United States of America*	[Level NA]<br><br>Qualitative study<br>*N*=18 (Young adults with a history of heavy drinking, ages 18-25 years old)	Objective included exploration of use-ability of Texting to Reduce Alcohol Consumption, an interactive text message intervention.	Results indicated that Texting to Reduce Alcohol Consumption could reduce heavy drinking in non-treatment-seeking young adults. The study identified four themes regarding user experiences with intervention: (1) ease of use, (2) comfort and confidentiality, (3) increased awareness of drinking behaviour, and (4) accountability for drinking behaviour.
Garnett, Crane, West, Brown, & Michie (2015)<br><br>*United Kingdom*	[Level V]	Objective included identifying the behaviour change techniques to be included in a smartphone	12 behaviour change techniques were identified as likely to be effective. As judged by

	N= 7 (International Academic Experts)	application to reduce alcohol consumption, using formal expert consensus methods.	experts, the results indicated that the behaviour change technique with the greatest potential to be included were goal-setting, self-monitoring, planning goals and feedback. The strategies most likely to keep users engaged were design, ease of use and unique tailoring of design.
Donoghue, Patton, Phillips, Deluca, & Drummond (2014)<br><br>*United Kingdom*	[Level I]<br><br>Meta-analysis	Objective included analysis of effectiveness of electronic screening and brief intervention with people who are non treatment-seeking at-risk drinkers.	In total, 23 studies were deemed eligible for review. 17 studies were finally included in the meta-analysis. The majority of the studies comprised of student populations. The study found that electronic screening with brief intervention was effective.
Bendtsen & Bendtsen (2014)<br><br>*Sweden*	[Level I]<br><br>Randomized Control Trial<br><br>*N*= 5499 (Swedish students)	Explored the acceptability and feasibility in a non-treatment-seeking group of university students of a fully automated alcohol intervention, comparing two modes of delivery by randomizing participants to receive the intervention either by text messaging or by email.	No significant difference was found regarding satisfaction with the length and frequency of the intervention, regardless of the mode of delivery.
Haug, Schaub, Venzin, Meyer, John, & Gmel (2013)<br><br>*Switzerland*	[Level III]<br><br><br>*N*= 364	Explored the effectiveness of individually tailored web- and text messaging-based intervention	The study revealed decreases in the percentage of persons with binge drinking from baseline to follow-up assessment ($P<.001$), the percentage of persons with alcohol-related problems ($P=.009$), and in the mean number of standard drinks per week: 13.4 (SD 15.3) to 11.3 (SD 14.0), $P=.002$. There was a decrease in the mean of the maximum number of drinks consumed on an occasion: 11.3 (SD 10.3) to 10.5 (SD 10.3), $P=.08$.

Beckjord & Shiffman (2014)<br><br>*United States of America*	[Level V]<br><br>Literature Review	Objective included comparing Ecological Momentary Assessment (EMA) and real-time intervention Ecological Momentary Intervention (EMI)	EMA and EMI are approaches that can extend the impact of behavioral intervention in managing binge drinking.
Gajecki, Berman, Sinadinovic, Rosendahl, & Andersson (2014)<br><br>*Sweden*	[Level I]<br>Randomized Control Trial<br><br>*N*=1932	Tested two smartphone applications targeting drinking choices, with the goal of reducing at-risk drinking among Swedish university students.	Results were not significant for primary outcome.
